# Plasma 8-OHdG act as a biomarker for steroid-induced osteonecrosis of the femoral head

**DOI:** 10.1186/s12891-023-06804-0

**Published:** 2023-10-12

**Authors:** Peng Peng, Mincong He, Weihua Fang, Mengqi Lai, Fangjun Xiao, Wei He, Huan Xiao, Qiushi Wei

**Affiliations:** 1 Guangzhou University of Chinese Medicine, Guangzhou, 510405 China; 2 Guangdong Research Institute for Orthopedics and Traumatology of Chinese Medicine, Guangzhou, 510378 China; 3 Department of Orthopaedics, The Third Affiliated Hospital, Guangzhou University of Chinese Medicine, Guangzhou, 510378 China; 4 Department of Orthopedics, Bijie Traditional Chinese Medicine Hospital, Bijie, 551700 China

**Keywords:** Steroid-induced osteonecrosis of the femoral head, 8-hydroxy-2'-deoxyguanosine (8-OHdG), Oxidative stress, Collapse

## Abstract

**Background:**

Oxidative stress was closely related to the occurrence and development of Steroid-induced osteonecrosis of the femoral head (SIONFH). 8-hydroxy-2'-deoxyguanosine (8-OHdG) is a important index of oxidative stress. The aim of this study is to investigate the role of 8-OHdG in the development of SIONFH.

**Methods:**

From May 2021 and November 2021, 33 patients diagnosed with SIONFH and 26 healthy controls were recruited in this study. Assessment included the radiography and pathology evaluation of clinical bone tissue, expression position and level of 8-OHdG, level of plasma 8-OHdG, as well as the receiver operating characteristic (ROC) curve.

**Results:**

We observed that expression levels of 8-OHdG in bone samples decreased with Association Research Circulation Osseous (ARCO) stages. Plasma 8-OHdG levels were significantly increased in the SIONFH group compared to the healthy control group. Plasma 8-OHdG level of pre-collapse patients was higher than that of post-collapse patients, the decreased plasma 8-OHdG level was related to higher ARCO stages.

**Conclusion:**

Plasma 8-OHdG may represent potential biomarkers during SIONFH at different stages. Higher plasma 8-OHdG levels indicated early stage of SIONFH. The current study provided new clues for early diagnosis and treatment for SIONFH.

## Introduction

Osteonecrosis of femoral head (ONFH) is a complex hip disease characterized by reduced blood supply to the femoral head, bone cell death, subchondral bone fractures, collapse of the femoral head, and joint dysfunction [1]. The annual prevalence of ONFH is about 20000 in the United States, and 75,000-150,000 in China [2, 3]. The exact causes of ONFH remain unclear, steroid usage is a common causative factor, accounting for 51% of ONFH cases [4]. Currently, the specific pathogenesis and molecular mechanism underlying steroid-induced ONFH (SIONFH) are not fully understood.

Accumulating evidences have shown that the high oxidative stress is related to the development of SIONFH [5–7]. Intracellular reactive oxygen species (ROS) has been widely studied as an important molecular in the process of oxidative stress. Previous studies have demonstrated that ROS take part in osteoclast differentiation as intracellular signal molecules by modulating receptor activator of NF-κB ligand (RANKL)-induced signalling [8, 9]. Chen et al. [10] reported that high oxidative stress following steroid administration may cause the decrease of the expression of antioxidant enzymes, which ultimately leads to osteoclast hyperactivity and subsequently progression of ONFH. Moreover, 8-hydroxy-2'-deoxyguanosine (8-OHdG) is an important index of oxidative stress, it formed through oxidation of guanine from damaged DNA [11, 12]. Until now, 8-OHdG has been widely used as biomarker for various disease, such as periodontal disease, atherosclerosis and cardiovascular disease [13, 14]. Fan et al. [15] have observed a significantly higher 8-OHdG expression in femoral head with SIONFH than in that of developmental dysplasia of the hip (DDH) patients and concluded that glucocorticoids induced oxidative stress and lead to osteocyte apoptosis in the process of SIONFH.

In the early stage of ONFH, active osteoclasts in necrotic zone may indicated the begining of repair. However, the persistent activation of osteoclasts results in bone resorption exceeds formation may lead to the loss of structural integrity and subchondral fracture [16]. Thus, the change of osteoclast activity may be correlated with collapse of ONFH. Here, we hypothesized that plasma 8-OHdG may be associated with disease progression in SIONFH. In the present study, we determined the correlation between plasma 8-OHdG concentrations and clinical features of different stages of the disease to investigate its roles in SIONFH.

## Materials and methods

### Study population

A total of 33 SIONFH patients were recruited from May 2021 to November 2021 from the Third Affiliated Hospital of Guangzhou University of Chinese Medicine. Twenty-six healthy volunteers were included as control group. The patients were diagnosed based on clinical evidence and radiological evidence of the hips. The history of steroid use was at least 2 g of prednisolone or its equivalent over a 3-month period, and the diagnosis must be made within 2 years of last steroid usage [17]. All of the patients were classified according to the ARCO staging system [18]. The inclusion criteria were as follows: a) radiographic criteria of ARCO stages ≥ II; and b) age above 18 years old. The exclusion criteria were as follows: a) patients who received previous treatments for avascular necrosis; b) patients with any diseases that could affect the hip joint; c) patients with severe metabolic disease. The control group included healthy volunteers who received medical examination at the same time. The healthy subjects had no history of steroid use or hip pain, and the anteroposterior and frog-leg lateral pelvic radiographs did not show any lesions. This study was approved by the ethics committee of The Third Affiliated Hospital of Guangzhou, University of Chinese Medicine. All participants provided written informed consent for inclusion in the study.

### Sample collection

Peripheral blood plasma was collected from 33 patients with SIONFH and 26 healthy volunteers, and stored at −80 °C until the day of measurement. Necrotic bone samples were obtained after total hip arthroplasty (THA) (*n* = 16 total). Bone samples in the control group (*n* = 6 total) from femoral neck fractures were obtained after THA. Bone samples were collected from normal tissue or subchondral necrotic zone with a depth of 1–4 mm from the cartilage. Bone samples were harvested and fixed in 4% formaldehyde for over 24h at room temperature.

### H&E staining of bone tissue

Bone samples from necrotic region and normal tissue from the control group were obtained and fixed in 4% formaldehyde. The samples were then decalcified, dehydrated, and inserted into paraffin wax to make 5-μm-thick sections. The tissue sections were stained by haematoxylin and eosin (H&E) and then observed using a microscope (3DHISTEC, Pannoramic MIDI). Ten HE staining images were randomly selected from each sample and the ratio of empty lacunae were calculated.

### Micro-CT scan

Micro-CT analyses were performed using a high-resolution device, NEMO Micro CT (NMC-200). The bone samples were placed in the chamber of a micro-CT set with the following parameters: 100-kVp X-ray source voltage and a resolution of 2μm Metrological analysis and 3D reconstruction were performed by Avatar 3.0. Structural parameters of trabecular bone were analyzed, including bone volume fraction (BV/TV), trabecular thickness (Tb.Th), trabecular number (Tb.N) and trabecular separation (Tb.Sp).

### Immunoshistochemistry for 8-OHdG

Five micron-thick tissue sections were dewaxed and rehydrated with different gradients of xylene and absolute ethanol. Antigen retrieval was performed using pH 6.0 citrate buffer. The 10% hydrogen peroxide was used to remove catalase, and then using 10% goat serum to block at room temperature for 1 hour. Then, the sections incubated with primary antibodies: rabbit anti-8-OHdG (1:1000; Proteintech Group, Inc). Specimens were incubated overnight at 4°C, then subjected to secondary antibody incubation (goat anti-rabbit immunoglobulin (IgG)-horseradish peroxidase HRP; 1:500 dilution; ZSGB-Bio, China) for 30 min at room temperature. After that, the sections were counterstained with haematoxylin and cover with mounting medium, observed under microscope (3DHISTEC, Pannoramic MIDI). The integrated optical density (IOD) values were quantified using Image-Pro Plus software version 6.0 (Media Cybernetics, Inc., Rockville, MD).

### Immunofluorescence for 8-OHdG

The 5-μm-thick sections were blocked in 5% BSA at 37°C for 1 h, then incubated with primary antibodies at 4°C overnight. Then, the sections were washed three times in PBS for 5 min each and incubated with corresponding secondary antibodies at 37°C for 2 h. The slices were stained with DAPI nuclear staining (Cell Signaling Technology, cat. #4083) for 1 min. Use the same antibody as for Immunoshistochemistry.

### Measurement of 8-OHdG concentrations

Plasma 8-OHdG was measured using a sensitive sandwich enzyme-linked immunosorbent assay (ELISA) according to the manufacturer’s instruction (Cusabio, MD, USA). 8-OHdG was used to generate a linear standard calibration curve (range 3.12-800 ng/ml). The manufacturer-reported precision was <12% (intra-assay) and <15% (inter-assay). The sensitivity of this assay was 3.12 ng/ml.

### Post hoc statistical power calculation

Statistic power (1-β) was calculated by Power and Sample Size Calculators (http://powerandsamplesize.com) to obtain the data of different mean 8-OHdG levels, standard error, and enrolled numbers of patients in each group [19]. Statistic power was regarded strong when > 0.8. The ratio between the sample sizes of the two groups is,

$$\mathrm{k}=\frac{{\mathrm{n}}_{\mathrm{A}}}{{\mathrm{n}}_{\mathrm{B}}}$$

The formula for calculation is as follows:

$${{n}_{A}=\mathrm{kn}}_{\mathrm{B }}\mathrm{and }{\mathrm{n}}_{\mathrm{B }}={\left(1+\frac{1}{\mathrm{k}}\right)\left(\upsigma \frac{{\mathcal{Z}}_{1-\mathrm{\alpha }/2}+{\mathcal{Z}}_{1-\upbeta }}{{\upmu }_{\mathrm{A }}-{\upmu }_{\mathrm{B}}}\right)}^{2}$$

$$1-\upbeta =\Phi \left(\mathcal{Z}-{\mathcal{Z}}_{1-\mathrm{\alpha }/2}\right)+\Phi \left(-\mathcal{Z}-{\mathcal{Z}}_{1-\mathrm{\alpha }/2}\right)$$

$$\mathcal{Z}=\frac{{\upmu }_{\mathrm{A }}-{\upmu }_{\mathrm{B }}}{\upsigma \sqrt{\frac{1}{{\mathrm{n}}_{\mathrm{A}}} + \frac{1}{{\mathrm{n}}_{\mathrm{B}}}}}$$

### Statistical analysis

SPSS version 23.0 (SPSS Inc., USA) was used to perform statistical analysis. The results are presented as mean ± standard deviation (mean ± SD). Data conforming to normal distribution were compared using Student’s t-test or one-way ANOVA, while those with non-normally distributed were tested using Mann-Whitney U test. *P* < 0.05 indicates statistical significance.

## Results

### Demography of cases in two groups

A total of 33 patients were recruited in the SIONFH group with a mean age of 38.19 ± 12.73 years. There were 26 patients in the control group with an average age of 40.15 ± 11.57 years. No significant difference in age was observed (Student’s T test, *P* > 0.05). There were 14 males and 19 females in the SIONFH group and 12 males and 14 females in the control group. There are 10 patients with stage II ONFH, 13 with III, and 10 with IV, respectively.

### Radiography and pathology evaluation of bone tissue

**Fig. 1:**
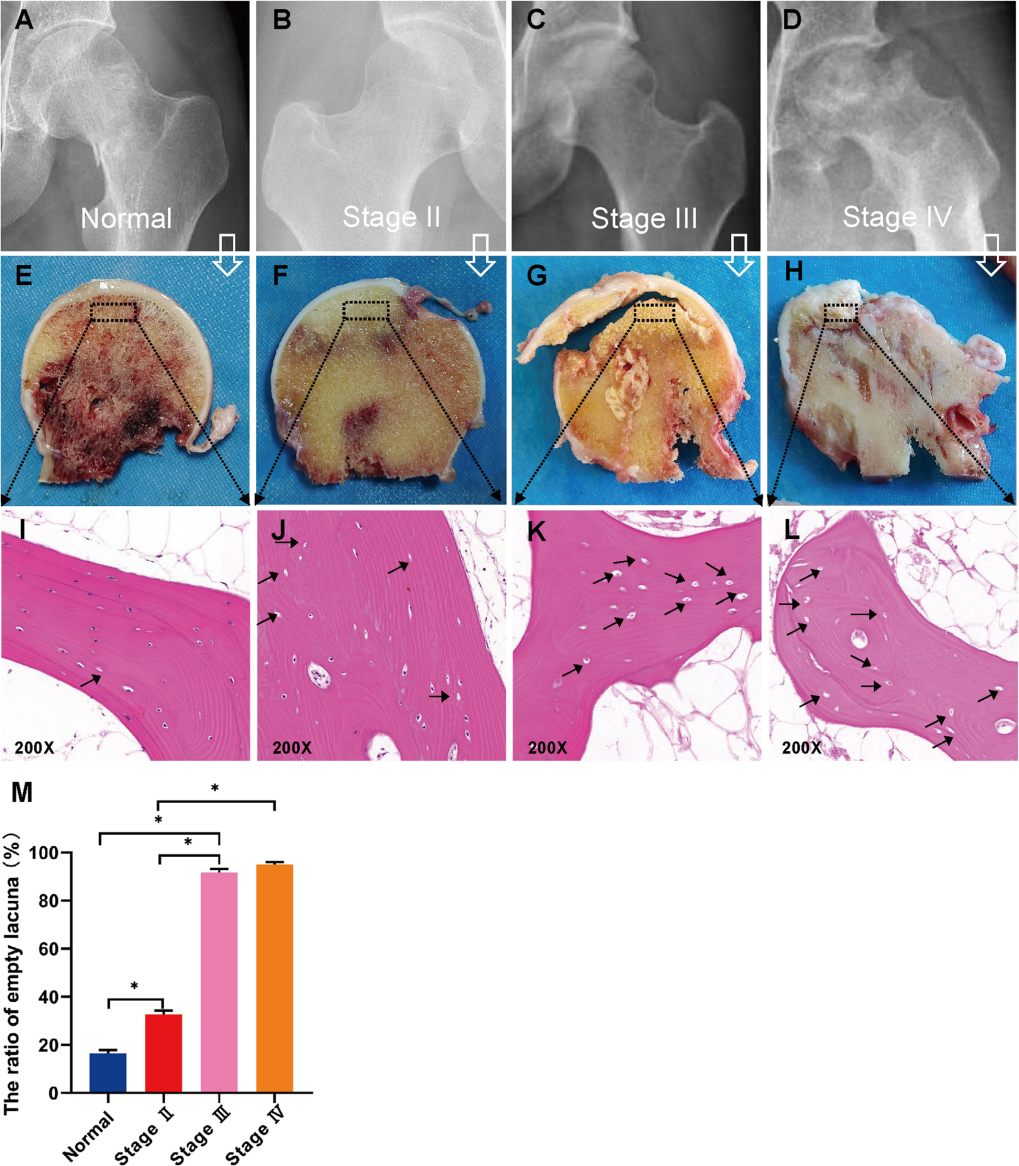
**A**-**D** X-ray images of control subject and SIONFH patients with different ARCO stages. **E**-**H** The macroscopic appearances of femoral head sections of control subject and SIONFH patients with different ARCO stages. The black dashed boxes indicated the regions collected for further analysis. **I**-**L** Histopathological features of control and SIONFH bone. **M** The ratio of empty lacunae in each SIONFH groups of different stage and control group. *P < 0.05

Figure 1A–D revealed the radiographic results of the control group and SIONFH group. Figure 1A illustrates a regular spherical femoral head and normal joint space. Figure 1B illustrates a Stage II necrotic femoral head with nonuniform density and the disappearance of local bone trabeculae. Figure 1C illustrates a typical stage III necrotic femoral head with the collapse of the articular surface. Figure 1D illustrates acetabulum changes, subchondral collapse and degenerative arthritis. Figure 1E–H show the macroscopic appearances of femoral head sections. No pathological sign was found in the homologous trabecular bone of the control group (Fig. 1E). Figure 1F shows the osteonecrotic bone within the subchondral area but the femoral head does not collapse. Figure 1G presents a distinct subchondral bone fracture with a rough cartilaginous surface. Figure 1H shows deteriorated and severely destroyed in the cartilage structure. Figure 1I–L shows the HE staining images of the femoral head sections. Figure 1I shows a healthy trabecular architecture with lots of osteocytes embedded. Figure 1J–L shows that with the stage increases, the osteocytes were absent in the lacunae and with an increasing number of empty lacunae. As shown in Fig. 1M, the ratio of empty lacunae in the SIONFH groups was significantly higher than those in the control group (*P* < 0.05). The ratio of empty lacunae in both stages III and IV were higher than that in Stage II (*P* < 0.05).

### Micro-CT scan

**Fig. 2:**
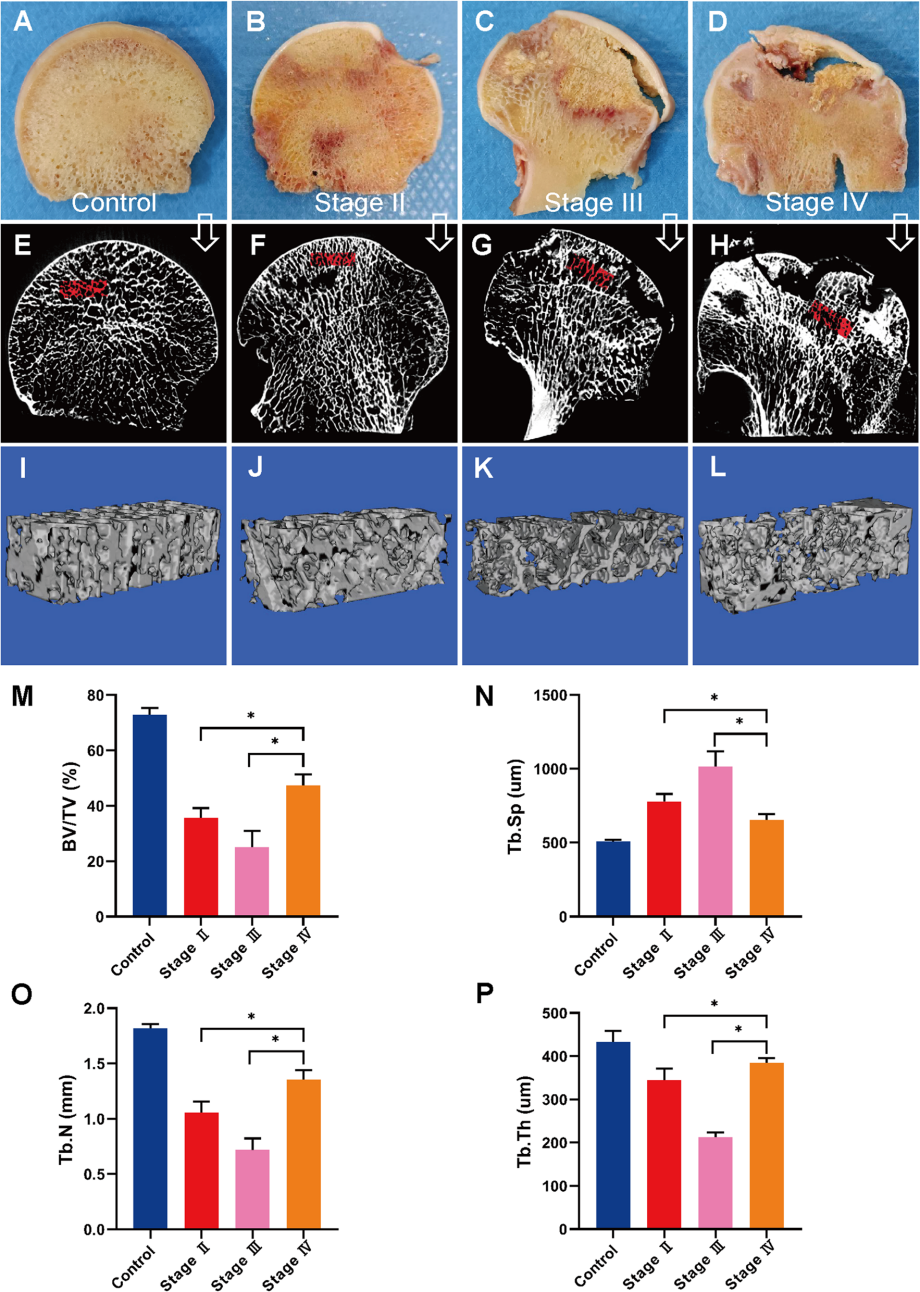
**A**–**D** General images of bone samples of control subject and SIONFH patients with different ARCO stages. **E**-**H** Micro-CT images of bone samples from the control subject and SIONFH patients with three ARCO stages. **I**-**L** The region of interest in bone samples from control and SIONFH bone samples. **M**-**P** Decreased value of BV/TV, Tb.Th and Tb.N and increased value of Tb.Sp were observed in stage III. Higher values of BV/TV, Tb.N, Tb.Th and lower values of Tb.Sp in Stage IV than that in Stage II were observed. *P < 0.05

Figure 2A–L shows the results of micro-CT images and evaluation. The structure characteristics of trabecular bone in necrotic area were significantly different in different stages (*P* < 0.05). We observed that decreased value of BV/TV, Tb.Th and Tb.N and increased value of Tb.Sp in Stage III. In addition, higher values of BV/TV, Tb.N, Tb.Th and lower values of Tb.Sp in Stage IV than that in Stage II were observed (Fig. 2M–P).

### Immunoshistochemistry for 8-OHdG

**Fig. 3:**
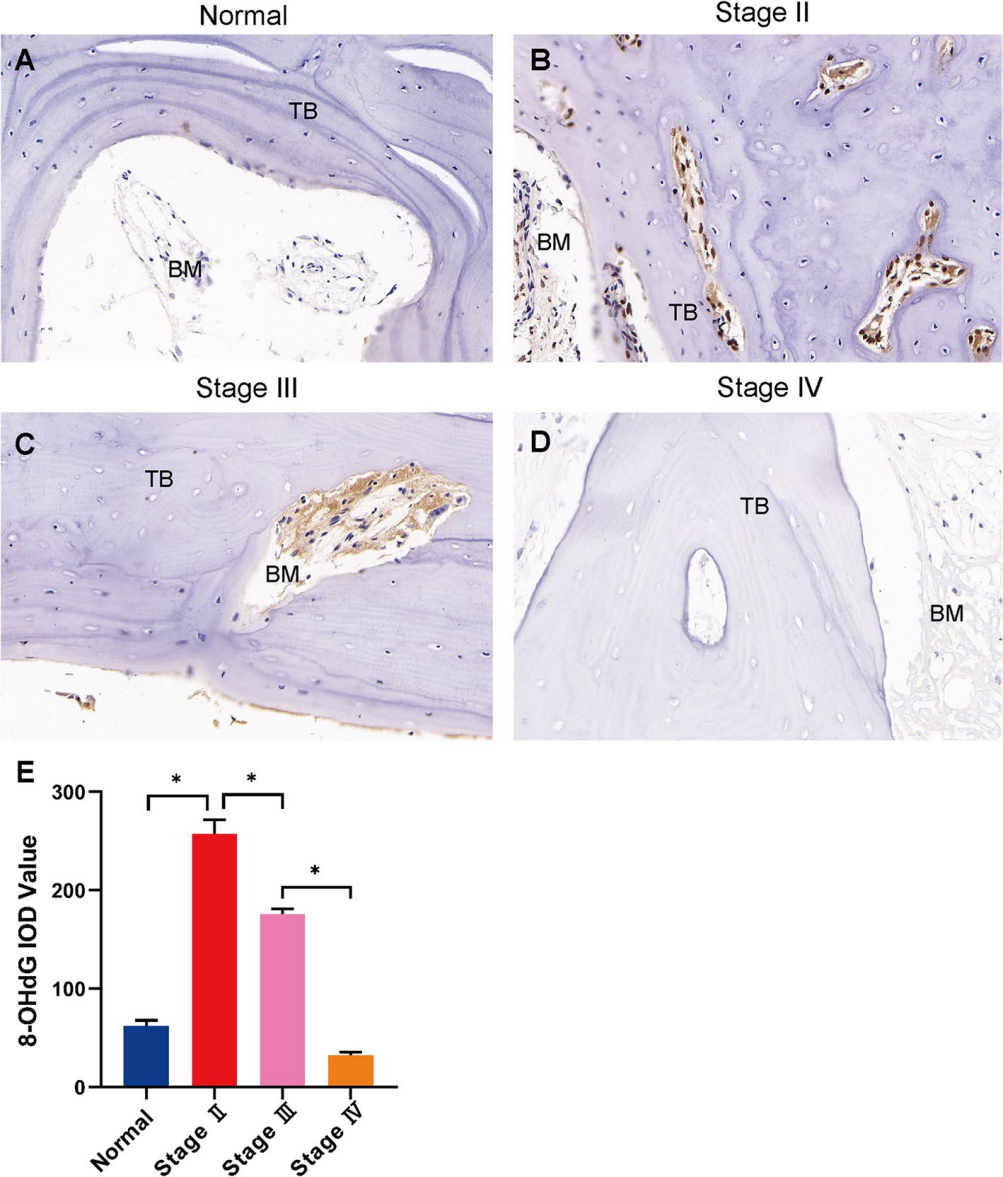
Immunohistochemistry results for 8-OHdG of bone samples in control group and SIONFH group with different stages. **A** An low-level presence of 8-OHdG were observed in healthy samples. **B**-**D** The levels of 8-OHdG was decreased as ARCO stage progress. **E** The IOD value of immunohistochemistry for 8-OHdG. *P < 0.05 TB: trabecular bone; BM: bone marrow

Positivity for 8-OHdG immunohistochemical staining was determined in both necrotic and healthy regions (Fig. 3). The 8-OHdG was detected on the trabecular bone, the osteoblast, the osteoclast, and bone marrow. An low-level presence of 8-OHdG were observed in healthy samples (*P* < 0.05) (Fig. 3A). The levels of 8-OHdG was decreased as ARCO stage progress (*P* < 0.05) (Fig. 3B-D). The quantitative result was shown in Fig. 3E.

### Immunofluorescence for 8-OHdG

**Fig. 4:**
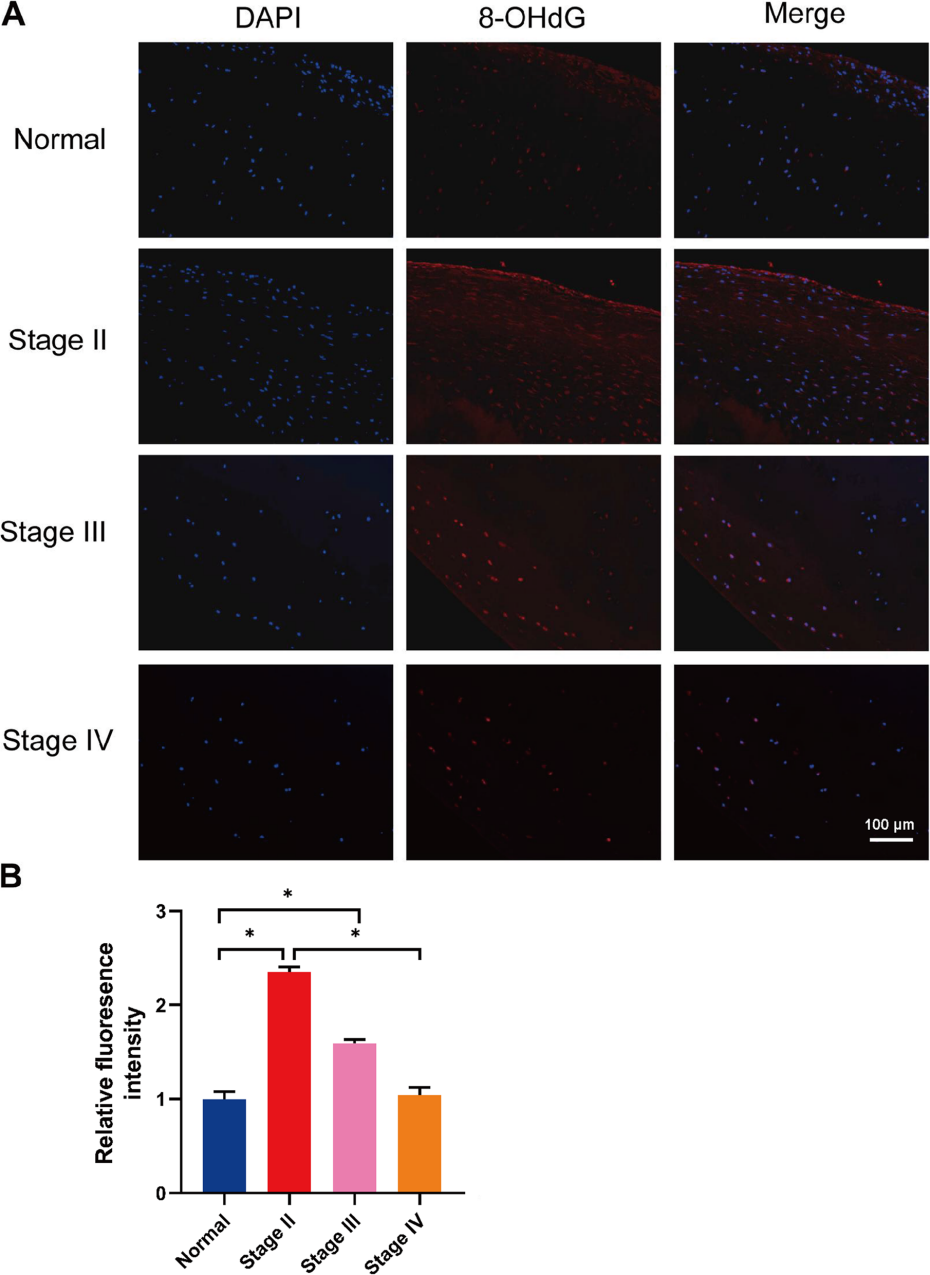
**A** Immunofluorescence expression of 8-OHdG. **B** Statistical analysis of immunofluorescence. *P < 0.05

Figure 4 shows the immunofluorescence expression of 8-OHdG. The results indicated that a low 8-OHdG expression was observed in the control group (Fig. 4A). Moreover, the expression levels of 8-OHdG decreased with ARCO stages (*P* < 0.05) (Fig. 4B).

### Plasma 8-OHdG levels in ONFH patients and control subjects

**Fig. 5:**
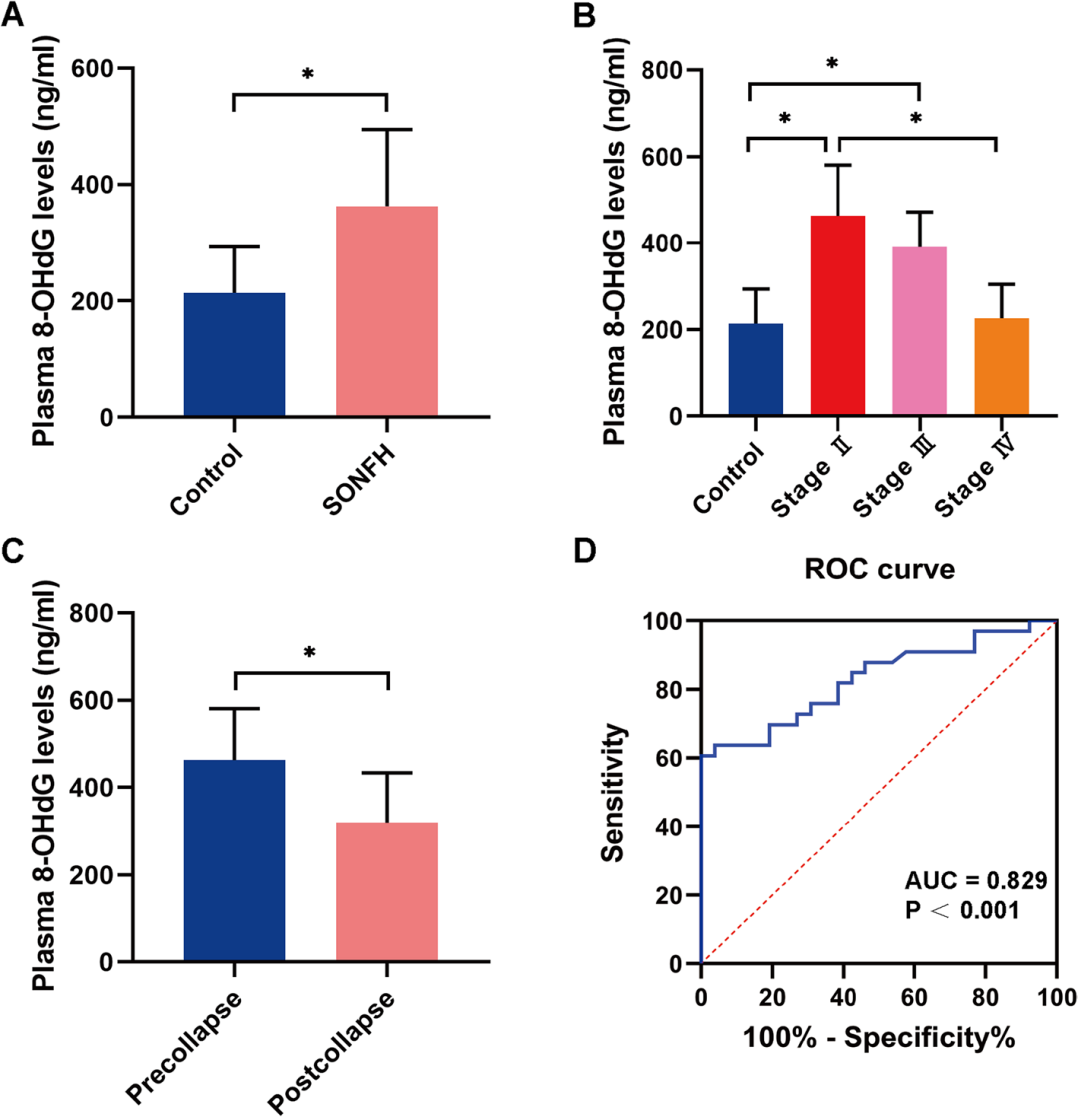
Overview shows plasma 8-OHdG level in SIONFH patients and control group. **A**-**C** Plasma 8-OHdG levels with statistical differences among different groups. **D** Receiver operating characteristic (ROC) curve and the area under the curve (AUC) in association with the sensitivity and specificity of SIONFH. *P < 0.05

The result of plasma 8-OHdG level from ELISA and relation between other clinical data was shown in Table 1. The plasma 8-OHdG level was 368.33 ± 131.92 ng/ml in the SIONFH group, which was significantly higher than 213.72 ± 80.15 ng/ml in the control group (*P* < 0.05) (Fig. 5A). Figure 5B revealed that plasma 8-OHdG levels were differ significantly with different ARCO stages among SIONFH patients (stage II: 481.52 ± 96.61 ng/ml, stage III: 390.95 ± 80.70 ng/ml, stage IV: 225.72 ± 79.28 ng/ml, *P* < 0.05). According to multi-ple comparisons, significantly differences were observed between stage II and stage III (*P* < 0.05), and between stage II and stage IV (*P* < 0.05). Moreover, the plasma 8-OHdG level of pre-collapse patients (481.52 ± 96.61 ng/ml) was higher than that of post-collapse patients (319.12 ± 114.62 ng/ml) (*P* < 0.05) (Fig. 5C). In addition, ROC curve analysis was also performed to investigate the predictive value of plasma 8-OHdG levels for SIONFH patients. The area under the curve (AUC) was 0.829 (*P* < 0.001), the sensitivity was 63.64%, and the specificity was 96.15% (cutoff, 321.4 ng/ml), which demonstrated that plasma 8-OHdG showed predictive value for diagnosis of ONFH (Fig. 5D).

**Table 1:** Plasma 8-OHdG levels in ONFH patients and control subject and potential relation between other clinical data

Group	Cases	8-OHdG level (pg/mL)	Comparison	*P* value
Control	26	213.72 ± 80.15	Control vs SIONFH	< 0.05
SIONFH	33	368.33 ± 131.92		
ARCO stages				
Stage II	10	481.52 ± 96.61	II vs III	0.023
Stage III	13	390.95 ± 80.70	III vs IV	< 0.05
Stage IV	10	225.72 ± 79.28	II vs IV	< 0.05
Precollapse	10	481.52 ± 96.61	Pre- vs. post-collapsed	< 0.05
Postcollapse	23	319.12 ± 114.62

### Statistical power

**Fig. 6:**
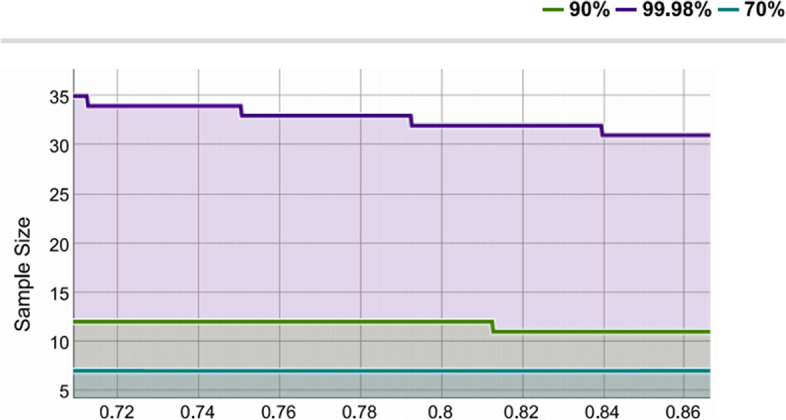
Statistic power determined by mean and sample size

After calculation, the statistical power was 0.99, suggesting that the sample size of 33 and the sampling ratio of 0.79 were sufficient to obtain the conclusion (Fig. 6).

## Discussion

In this study, we evaluated the relationship between plasma 8-OHdG level and the occurrence of SIONFH as well as disease process in patients with SIONFH. We found that in both plasma and protein level, 8-OHdG was significantly increased in the SIONFH group compared to the control group. The plasma 8-OHdG level of pre-collapse patients was higher than that of post-collapse patients, the decreased plasma 8-OHdG level was related to higher ARCO stages. The finding indicates that 8-OHdG could possibly be used as a biomarker to assess the progress of ONFH.

Currently, the specific pathogenesis and molecular mechanism of SIONFH is complex and remain unclear. Increasing studies indicated that oxidative stress was closely related to the occurrence and development of SIONFH. It has been reported that excessive usage of glucocorticoids could lead to oxidative injury [15, 20]. Oxidative stress is defined as the imbalance between prooxidants and antioxidants in the organism which caused by an augmented level of ROS, free radicals, and other reactive molecular species [21, 22]. Pathological excessive generation of ROS can result in oxidative injury, which is related to osteoarthritis, osteoporosis and SIONFH [23, 24]. The current understanding of ROS mediated oxidative stress in the process of SIONFH is that oxidative stress may enhance the osteoclast activity and induced osteoblast and osteocyte apoptosis [10, 15]. ROS take part in osteoclast formation and resorptive activity as intracellular signal molecules by supporting the activation of RANKL-induced signalling [25, 26]. Antioxidant therapy may be a promising method to prevent the process of SIONFH by inhibiting ROS level [10]. Besides, ROS mediated oxidative stress can induce cell apoptosis of osteocyte and osteoblast [27, 28].

8-OHdG, one of the most pivotal markers of oxidative stress, has been used to measure the effect of endogenous oxidative damage to DNA [29]. Recently, 8-OHdG has been used as biomarker for oxidative stress in various fields, such as atopic diseases in children, depression in bipolar disorder, and a variety of cancers [12, 30, 31]. An experimental study in rats showed that the 8-OHdG immunoreactivities in the vessels, adipocytes, bone marrow, and trabeculae increased significantly in the SIONFH group compared to the control group, indicating an increased oxidative injury in SIONFH model [32]. In theory, the secretion of 8-OHdG should be increased following the oxidative stress caused by SIONFH. Here, we suppose that the increased 8-OHdG level accompanied by the oxidative stress might be an important pathological process of SIONFH. In this study, we observed that plasma 8-OHdG level was significantly increased in the SIONFH patients compared to the control group. This results indicated that increased plasma 8-OHdG levels are associated with the occurrence of SIONFH and might act as a predictive factor for SIONFH diagnosis.

The imbalance of osteoblast and osteoclast activity also plays a key role in the progression of SIONFH and the collapse of femoral head. Our previous study has been demonstrated that osteoblast and osteoclast activity changed during the progression of SIONFH in necrotic area in femoral heads [33]. In the early stages of SIONFH, osteoclast activity is significantly increased, and excessive osteoclast activity is considered to be the cause of femoral head collapse. In the late-stage SIONFH, it can be observed that osteoclast activity decreased and osteoblast activity increased in necrotic femoral heads. The results of micro-CT also demonstrated that a higher osteoclastic level in Stage II and III could weaken the bone microstructure in trabecular scale, which might lead to the collapse of femoral heads. With the progress of SIONFH to the stage IV, trabecular quantity increased as bone reconstruction took place. As previously mentioned, ROS mediated oxidative stress may affect the differentiation of osteoblast and osteoclast. Therefore, we further investigated the relation between SIONFH and 8-OHdG levels in tissue and plasma, especially in different stages of SIONFH. Patients with ARCO II and III had significantly higher plasma 8-OHdG levels compared with Stage IV patients and the control group. The protein expression levels of 8-OHdG in femoral head bone samples were consistent with plasma levels. A higher-level protein expression of 8-OHdG were observed with SIONFH samples than in that of healthy samples, these results are similar with previous study [15]. These findings suggested that increased plasma 8-OHdG levels are related to the process of collapse. Increased expression of 8-OHdG followed by oxidative stress which may indicates an enhance of osteoclast activity and the decrease of osteoblast activity in the early stages. This change alters the balance between osteoblast and osteoclast activity resulting in the collapse of femoral head. Several studies suggested that oxidative injuries usually present following steroid administration at the early-stage ONFH [34, 35], these results are consistent with the phenomenon we observed in the present study. Meanwhile, 8-OHdG expression decreased in the late stages revealed that osteoclast activity was decreased in the late stages of SIONFH. In addition, the results of X-ray, macroscopic appearances of femoral head sections as well as the HE staining indicated that the pathological features of SIONFH are apoptosis of osteocytes and rising number of lacunae, which proved that the samples harvested were reliable and can reflect the pathological changes in different stages.

This is the first study demonstrated the association of 8-OHdG expression with the disease severity of SIONFH. However, several limitations needed to be acknowledged in our study. First, it is difficult to enroll patients at an early stage of ONFH, so that we did not evaluated the levels of 8-OHdG in stage I which makes it hard to predict the early stage of ONFH. Second, the control group was not matched with glucocorticoid consumption, only with age and sex. Third, the number of samples in this study was limited, and larger samples are needed for further study.

In conclusion, plasma 8-OHdG may represent potential biomarkers during SIONFH at different stages. Higher plasma 8-OHdG levels indicated early stage of SIONFH. The current study provided new clues for early diagnosis and treatment for SIONFH.

## Data Availability

Data will be made available from the corresponding author on request.

## Abbreviations

SIONFH: Steroid-induced osteonecrosis of the femoral head
8-OHdG: 8-hydroxy-2'-deoxyguanosine
ONFH: Osteonecrosis of femoral head
ROS: Reactive oxygen species
ARCO: Association Research Circulation Osseous
THA: Total hip arthroplasty
